# Persistent health inequalities over 20 years among adults with intellectual disabilities who display behaviours that challenge: evidence from English primary care records

**DOI:** 10.1192/bjo.2026.11043

**Published:** 2026-05-26

**Authors:** Memta Jagtiani, Aws Sadik, Louise Marston, Shoumitro Deb, Dheeraj Rai, Bhathika Perera, Rohit Shankar, Juliette A. O’Connell, Angela Hassiotis

**Affiliations:** Division of Psychiatry, University College London, UK; Department of Political Science, University College London, UK; Centre for Academic Mental Health and MRC Integrative Epidemiology Unit, University of Bristol, UK; Department of Primary Care and Population Health, University College London, UK; Department of Brain Sciences, Imperial College London, UK; Bristol Medical School, University of Bristol, UK; Peninsula Medical School, Plymouth University, UK; School of Pharmacy and Pharmaceutical Sciences, Trinity College Dublin, Ireland

**Keywords:** Behaviours that challenge, intellectual disability, mental health services, neurodevelopmental disorders, longitudinal data

## Abstract

**Background:**

Adults with intellectual disabilities who display behaviours that challenge (BtC) are more prone to poor health.

**Aims:**

This study seeks to evidence the long-term health outcomes for those who display BtC.

**Method:**

We conducted a longitudinal cohort study of adults with intellectual disabilities aged ≥18 years in England, using data from the Clinical Practice Research Datalink Aurum (January 2003 to December 2023) linked to Hospital Episode Statistics and Office for National Statistics. Main outcome measures were annual health checks, general practitioner referrals, emergency visits, out-patient attendance, in-patient admissions and mortality.

**Results:**

Among 83 166 adults with intellectual disabilities (mean age 38.6 years), 18.5% had a record of BtC, with similar sociodemographic distributions to those without BtC, but higher rates of physical and mental health comorbidities and uptake of annual health checks. A total of 72.5% of participants with BtC were receiving psychotropic medication(s). Adults with BtC had higher rates of mental health out-patient attendance (odds ratio: 1.42, 95% CI 1.33–1.52) and in-patient admissions (incidence rate ratio (IRR): 1.19, 95% CI 1.09–1.29), but consistently lower rates of physical health out-patient attendance (IRR = 0.81, 95% CI 0.78–0.84) and in-patient admissions (IRR = 0.77, 95% CI 0.74–0.79), after adjusting for demographic and clinical characteristics. BtC were not associated with mortality after adjustment for comorbidities (hazard ratio: 0.97, 95% CI 0.93–1.00).

**Conclusions:**

This longitudinal study not only corroborated the markedly elevated burden of physical and mental health comorbidities among individuals displaying BtC, but also indicated that repeated efforts to improve health outcomes have yielded minimal measurable benefit over time. The apparent absence of progress is likely underpinned by a combination of insufficiently effective or poorly tailored interventions and wider systemic constraints that limit the capacity of services to respond to the complex needs of this population.

Intellectual disability is a neurodevelopmental disorder characterised by significant limitations in intellectual functioning and adaptive behaviour, with onset during the developmental period.^1^ Individuals with intellectual disabilities often display behaviours that challenge (BtC), including verbal or physical aggression, self-injury and destruction to property. These behaviours are associated with poor quality of life, social exclusion and increased risk of psychiatric hospital admissions.^2^

Multiple interventions including psychotropic medications and psychosocial interventions have been trialled in the management and prevention of BtC. In a meta-analysis of 82 reports of clinical trials, Groves et al found no superiority of pharmacological versus non-pharmacological interventions for BtC and its specific typologies.^3^ Psychotropic medications, especially antipsychotics, are frequently prescribed off-licence to manage BtC, particularly where there may be risk to self or others.^4,5^ In England, national guidelines recommend that antipsychotics be used only in the short term when risks are high, and alongside non-pharmacological interventions.^6^ However, in practice, adults with intellectual disabilities who display BtC are more likely to be prescribed antipsychotics long term,^7^ the drivers of which are underpinned by multiple factors such as clinical presentation, efficacy of service approaches or even caregiver preferences.^8,9^ Since 2008, national initiatives such as annual health checks (AHCs) for people with intellectual disabilities aged 14 years and over have aimed to improve the mental and physical health of this population and reduce health inequalities.^10^ Buszewicz et al found that AHCs were associated with improved detection of health conditions, increased delivery of health promotion and reduced preventable hospital admissions.^11^

## Gaps in literature

Longitudinal associations between BtC, therapeutic input and health outcomes among adults with intellectual disabilities remain under-researched.^12^ Given substantial uncertainties of the long-term outcomes of the relapsing-remitting nature of BtC, sources such as large routinely collected data may offer a unique opportunity to assess outcomes over time, generate system-level insights into patterns of care, and monitor prescribing practices and potential inequalities within this vulnerable group.

## Aims

We aimed to investigate and compare clinical outcomes across primary and secondary healthcare use in adults with intellectual disabilities with and without BtC, aged 18 years and over. Our objectives were as follows:

- to describe the cohort of adults with intellectual disabilities in terms of sociodemographic and clinical characteristics;
- to estimate the prevalence of psychotropic prescribing and non-pharmacological interventions in the identified cohort;
- to estimate and compare patterns of AHCs and general practitioner (GP) referrals for further assessment and/or treatment;
- to determine the proportion of individuals with and without BtC identified in the cohort who subsequently had any hospital contact, i.e. attendance and admissions;
- to examine whether BtC is associated with mortality and any hospital contact, i.e. attendance and admissions, alongside adjusting for age, ethnic group, deprivation index and diagnostic comorbidities.

We hypothesised that individuals with BtC would have higher mortality, higher rates of hospital attendance and admissions, poorer physical health profiles and greater uptake of AHCs compared with those without BtC.

## Method

### Participants

We identified a longitudinal cohort of patients with intellectual disabilities who were registered with a primary care practice in England between 2003 and 2023, from the Clinical Practice Research Datalink (CPRD) Aurum. The CPRD Aurum is representative of the population^13^ and contains electronic medical records for over 16 million patients.^14^

Relevant SNOMED CT codes related to intellectual disabilities, BtC, health comorbidities and neurodevelopmental or psychiatric conditions were first identified using CPRD Aurum’s medical code dictionary. These codes were then cross-checked against existing code lists developed by other research groups and institutions (e.g. LSHTM Data Compass; University of Exeter: https://github.com/Exeter-Diabetes/CPRD-Codelists). The final code lists (available on request) were agreed through review and verification by clinicians within the research team.

The eligibility criteria for cohort entry were having a record of intellectual disabilities and linked data (see below), registered with an up-to-standard general practice and aged 18 years or older at or after 1 January 2003 until 31 December 2022, to allow for at least a year of follow-up. Individual patient records were analysed from cohort inception or registration start date or year they turned 18 years old (whichever was the latest), until the end of data study: 31 December 2023, registration end date, patient death date or the year they turned 100 years old (whichever was the earliest).

### Data linkage

In addition to the CPRD Aurum, this study used linked data from Office for National Statistics (ONS) death registration data and Hospital Episode Statistics (HES) secondary care data-sets, including HES Admitted Patient Care (HES APC), HES Outpatient (HES OP) data and HES Accident and Emergency (HES A&E) data. All of the linked data-sets covered the years of the cohort (2003 to 2023), except HES A&E data, which only covered the period 2007 to 2020. It was replaced by the Emergency Care Data Set from April 2020, but is not yet linked to CPRD. HES data is not available for everyone as HES does not link to all practices, therefore the cohort size is smaller after data linkage. Linkage of CPRD to HES data-sets is carried out by a trusted third party (NHS Digital) to maintain patient confidentiality.

### Ethics statement

The authors assert that all procedures contributing to this work comply with the ethical standards of the relevant national and institutional committees on human experimentation and with the Declaration of Helsinki of 1975, as revised in 2013 (https://www.wma.net/policies-post/wma-declaration-of-helsinki/). All procedures involving human patients were approved by the Health Research Authority to support research using anonymised patient data (reference: 21/EM/0265).

### Consent statement

The CPRD electronic Research Application Portal was used to request access to the CPRD data and obtain approval for the study (protocol reference: 23_002605). Patients can opt out of having their de-identified data shared via CPRD. Similarly, all linked data-sets used the opt-out consent model.

### Exposure

The exposure for this study was adults with intellectual disabilities who display BtC. Individuals without BtC served as the comparison group, enabling assessment of differences in physical and mental health outcomes associated with BtC.

### Outcomes

We were interested in (a) AHCs, (b) GP referrals, (c) emergency visits (HES A&E), (d) physical and mental health out-patient attendance (HES OP), (e) physical and mental health in-patient admissions (HES APC) and (f) all-cause mortality (CPRD-ONS).

The segregation of physical and mental health in-patient admissions and outpatient attendance was achieved by grouping the ICD-10 codes, where codes that started with F indicated mental ill-health and all other codes were classified as physical ill-health. GP referrals were categorised as physical health referral, mental health referral or unspecified referral. The most used codes for the latter are ‘referred to hospital’ or ‘hospital referral’.

### Potential confounders

We included gender, ethnicity and Index of Multiple Deprivation (IMD) quintile as potential sociodemographic confounders. Gender and ethnicity were reported as recorded in the patient’s electronic health record. Ethnicity, recorded in both CPRD and HES, was reported as defined in either source of data at any time point^15^ and grouped according to the UK 2011 Census Ethnic Group categories:^16^ Asian, Black, Mixed, White or Other. Where conflict existed between CPRD and HES, we prioritised the CPRD classification because HES ethnicity recording is less accurate.^17^ IMD was based on patient postcode and, where this was missing, the primary care practice postcode was used to indicate IMD quintile.

We investigated mental and physical comorbidities as potential confounders as they are key drivers of mortality and hospital admissions.

### Statistical analysis

We conducted descriptive analyses to characterise the study cohort in terms of demographic variables, IMD, physical and mental health comorbidities, and the types of interventions received.

The cohort was stratified by the presence or absence of BtC. Categorical variables were summarised with frequencies and percentages, whereas continuous variables such as age and follow-up time were described with means and standard deviations. Follow-up time was calculated from the date of cohort entry to when the participant left the cohort. Patient-years were computed to reflect the total time at risk contributed by individuals over the follow-up period. The proportion of patients who received no intervention, non-pharmacological interventions only (e.g. behavioural or psychological interventions – code lists available on request), psychotropic medications only (antidepressants, antipsychotics, anxiolytics, sedatives and hypnotics, anti-Parkinsonian agents, mood stabilisers, attention-deficit hyperactivity disorder (ADHD) drugs and anti-dementia drugs), and both types of interventions at any time were also computed.

For all outcomes, we included the same variables in the same order, adjusting for demographic (age, gender, ethnicity, IMD quintile) and clinical status (neurodevelopmental conditions, mental and physical health comorbidities). Some data were missing for ethnicity and IMD. All analyses used complete-case analysis and were conducted using R version 4.2.5 for Windows (Posit PBC, Boston, Massachusetts, USA; https://posit.co/download/rstudio-desktop).

#### Mortality

We used Cox proportional hazards regression to assess the relationship between BtC and all-cause mortality. Time-to-event was calculated from age 18 for all to date of death or where participants were censored. We reported hazard ratios and corresponding 95% confidence intervals. Kaplan–Meier survival curves were plotted to visualise unadjusted survival between BtC and non-BtC groups.

#### Service use

For physical health out-patient attendance and physical and mental health hospital admissions, we applied negative binomial regression models because of overdispersion in the outcomes, with an offset of 1/time in the cohort to account for the different lengths of time participants were in the cohort. We reported the incidence rate ratios and corresponding 95% confidence intervals.

We used logistic regression to model the odds of having at least one mental health out-patient attendance as a binary outcome because there were few people with mental health out-patient attendance recorded, and the negative binomial models did not converge. We reported the odds ratios and corresponding 95% confidence intervals.

## Results

### Description of the cohort

**Fig. 1:**
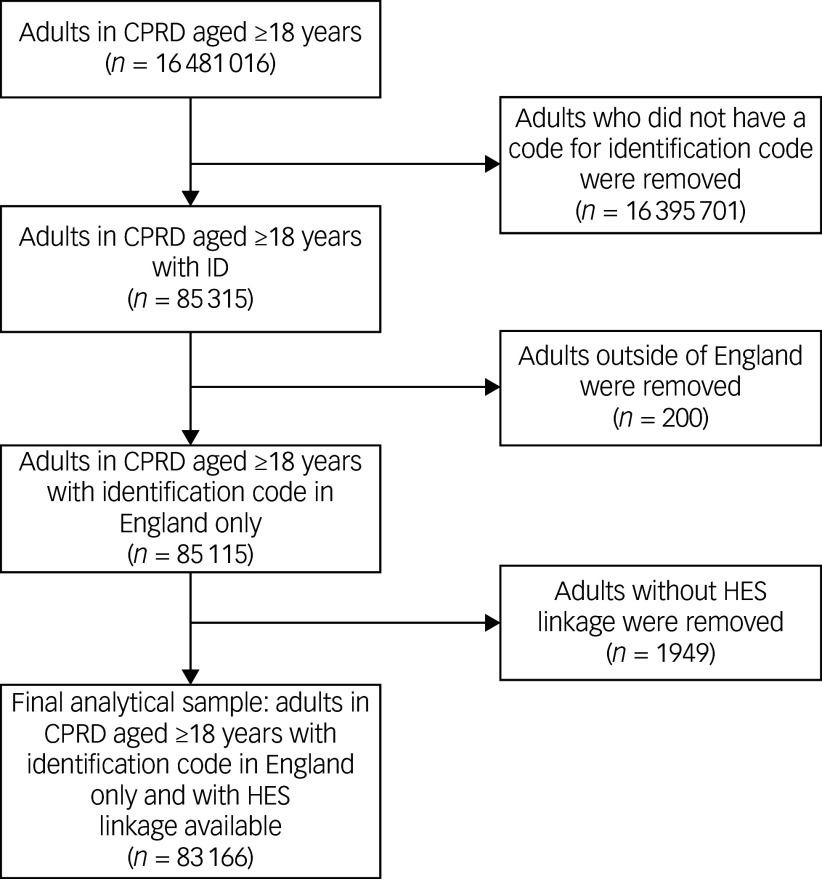
Flow diagram of participants. CPRD, Clinical Practice Research Datalink; ID, intellectual disabilities; HES, Hospital Episode Statistics.

Figure 1 illustrates how the final analytical sample (*N* = 83 166) was derived.

The total number of patient-years was 540 509, with a mean follow-up of 6.5 years (s.d. = 5.0). The mean age at cohort entry was 38.6 years. Out of the total cohort (*N* = 83 166), 18.5% (*n* = 15 368) had a record of BtC. Men made up 57.3% of the cohort, with similar gender distributions across BtC and non-BtC groups. Most of the cohort was White (82.6%). The distribution across IMD quintiles was comparable between groups, with 23.9% (*n* = 19 845) residing in the most deprived areas (Table 1).

**Table 1:** Sociodemographic and clinical profile of the cohort with intellectual disabilities from 2003 to 2023

Characteristics	Overall, *n* (%)	BtC, *n* (%)	No BtC, *n* (%)
Cohort, *n* (row %)	83 166 (100.0)	15 368 (18.5)	67 798 (81.5)
Gender, *n* (column %):			
Female	35 480 (42.7)	6365 (41.4)	29 115 (42.9)
Male	47 686 (57.3)	9003 (58.6)	38 683 (57.1)
All-cause mortality			
Died between 1 January 2004 and 31 December 2023	22 933 (27.6)	5144 (33.5)	17 789 (26.2)
Age at death, years, mean (s.d.) for those who died	62.8 (16.0)	64.1 (14.8)	62.5 (16.3)
Age at death, years, median for those who died	64	65	64
Age			
Age at entry, mean (s.d.), years	38.6 (17.8)	41.2 (17.7)	38.1 (17.8)
Ethnicity			
White	68 693 (82.6)	13 247 (86.2)	55 446 (81.8)
Black	2957 (3.6)	549 (3.6)	2408 (3.6)
Asian	3158 (3.8)	594 (3.9)	2564 (3.8)
Mixed and Other	1288 (1.5)	190 (1.2)	1098 (1.6)
Missing	7070 (8.5)	788 (5.1)	6282 (9.3)
Index of Multiple Deprivation			
1 – Least deprived	8736 (10.5)	1534 (10.0)	7202 (10.6)
2	11 675 (14.0)	2293 (14.9)	9382 (13.8)
3	14 221 (17.1)	2811 (18.3)	11 410 (16.8)
4	16 026 (19.3)	2880 (18.7)	13 146 (19.4)
5 – Most deprived	19 845 (23.9)	3479 (22.6)	16 366 (24.1)
Missing	12 663 (15.2)	2371 (15.4)	10 292 (15.2)
Physical health comorbidities			
High cholesterol	24 403 (29.3)	6070 (39.5)	18 333 (27.0)
Constipation	16 084 (19.3)	5331 (34.7)	10 753 (15.9)
Type 2 diabetes	8070 (9.7)	1830 (11.9)	6240 (9.2)
Epilepsy	17 281 (20.8)	4707 (30.6)	12 574 (18.5)
High blood pressure	20 290 (24.4)	4878 (31.7)	15 412 (22.7)
Overweight or obese	27 739 (33.4)	6920 (45.0)	20 819 (30.7)
Severe mobility difficulties	9388 (11.3)	3309 (21.5)	6079 (9.0)
Mental health comorbidities			
Anxiety	9434 (11.3)	3097 (20.2)	6337 (9.3)
Lifetime record of dementia	4250 (5.1)	1331 (8.7)	2919 (4.3)
Depression	14 458 (17.4)	5605 (36.5)	8853 (13.1)
Severe mental illness			
Lifetime record of bipolar disorder	16 927 (20.4)	6411 (41.7)	10 516 (15.5)
Lifetime record of psychoses or schizophrenia	7413 (8.9)	2753 (17.9)	4660 (6.9)
Neurodevelopmental disorders			
Lifetime record of ADHD	4402 (5.3)	1056 (6.9)	3346 (4.9)
Lifetime record of autism	12 221 (14.7)	3123 (20.3)	9098 (13.4)
Interventions			
None	33 567 (40.4)	2486 (16.2)	31 081 (45.8)
At least one non-pharmacological intervention only	1301 (1.6)	266 (1.7)	1035 (1.5)
At least one psychotropic medication only	44 741 (53.8)	11 144 (72.5)	33 597 (49.6)
Both	3557 (4.3)	1472 (9.6)	2086 (3.1)

BtC, behaviours that challenge; ADHD, attention-deficit hyperactivity disorder.

The BtC group had higher rates of physical comorbidities than the non-BtC group in all of the included conditions, i.e. cholesterol (39.5% *v*. 27.0%), constipation (34.7% *v*. 15.9%), type 2 diabetes (11.9% *v*. 9.2%), epilepsy (30.6% *v*. 18.5%), high blood pressure (31.7% *v*. 22.7%), overweight or obesity (45.0% *v*. 30.7%), and severe mobility difficulties (21.5% *v*. 9.0%). A similar pattern was observed for mental health conditions, with notably higher prevalence of anxiety (20.2% *v*. 9.3%), depression (36.5% *v*. 13.1%), bipolar disorder (41.7% *v*. 15.5%), psychosis or schizophrenia (17.9% *v*. 6.9%), ADHD and autism (6.9% *v*. 4.9% and 20.3% *v*. 13.4%, respectively) in the BtC group.

Mortality was higher among those with BtC (33.5% *v*. 26.2%), as was the mean age at death compared with the non-BtC group (64.1 *v*. 62.5).

More than half of the cohort (53.8%) were recorded as receiving at least one psychotropic medication only, with this being especially common in the BtC group, where 72.5% were prescribed any psychotropic medication. Notably, 40.4% of the cohort did not receive psychotropic medication(s) or non-pharmacological intervention(s). In the BtC group, 9.6% were recorded as receiving both psychotropic medication(s) and non-pharmacological intervention(s), compared with 3.1% in the non-BtC group.

Stratified by intervention type (Table 2), those in the BtC group have higher mortality if receiving both interventions (37.0%) or at least one psychotropic medication (34.8%), compared with those receiving no intervention (26.3%) or at least one non-pharmacological intervention (25.1%). Between men and women, the largest difference was observed among those who had a record of at least one non-pharmacological intervention (65% men *v*. 35% women). There was little difference between ethnicities in terms of interventions received.

**Table 2:** Sociodemographic and clinical profile of the BtC cohort (*n* = 15 368), by intervention type

Characteristics	None	At least one non-pharmacological intervention only	At least one psychotropic medication only	Both
	*n* (%)	*n* (%)	*n* (%)	*n* (%)
	2486 (100)	266 (100)	11 144 (100)	1472 (100)
All-cause mortality				
Died between 1 January 2004 and 23 December 2023	655 (26.3)	64 (25.1)	3881 (34.8)	544 (37.0)
Age at death, years, mean (s.d.) for those who died	63.0 (17.6)	65.3 (13.0)	62.4 (14.8)	62.7 (14.6)
Age at death, years, median for those who died	63	67	63	64
Gender				
Female	901 (36.2)	93 (35.0)	4714 (42.3)	657 (44.6)
Male	1585 (63.8)	173 (65.0)	6430 (57.7)	815 (55.4)
Age				
Age at entry, years, mean (s.d.)	37.1 (18.9)	37.4 (18.1)	42.1 (17.5)	42.1 (16.9)
Ethnicity				
White	2,029 (81.6)	224 (84.2)	9694 (87.0)	1300 (88.3)
Black	132 (5.3)	12 (4.5)	354 (3.2)	51 (3.5)
Asian	145 (5.8)	15 (5.6)	375 (3.4)	59 (4.0)
Mixed and Other	41 (1.6)	4 (1.5)	132 (1.2)	13 (0.9)
Missing	139 (5.6)	11 (4.1)	589 (5.3)	49 (3.3)
Index of Multiple Deprivation				
1 – Least deprived	243 (9.8)	27 (10.2)	1105 (9.9)	159 (10.8)
2	404 (16.3)	39 (14.7)	1638 (14.7)	212 (14.4)
3	451 (18.1)	51 (19.2)	2050 (18.4)	259 (17.6)
4	487 (19.6)	58 (21.8)	2029 (18.2)	306 (20.8)
5 – Most deprived	587 (23.6)	53 (19.9)	2530 (22.7)	309 (21.0)
Missing	314 (12.6)	38 (14.3)	1792 (16.1)	227 (15.4)
Physical health comorbidities				
High cholesterol	699 (28.1)	88 (33.1)	4564 (41.0)	719 (48.8)
Constipation	503 (20.2)	72 (27.1)	4027 (36.1)	729 (49.5)
Type 2 diabetes	284 (11.4)	38 (14.3)	1317 (11.8)	191 (13.0)
Epilepsy	453 (18.2)	44 (16.5)	3653 (32.8)	557 (37.8)
High blood pressure	622 (25.0)	93 (35.0)	3591 (32.2)	572 (38.9)
Overweight or obese	1000 (40.2)	170 (63.9)	4884 (43.8)	866 (58.8)
Severe mobility difficulties	392 (15.8)	79 (29.7)	2272 (20.4)	566 (38.5)
Mental health comorbidities				
Anxiety	144 (5.8)	13 (4.9)	2546 (22.8)	394 (26.8)
Lifetime diagnosis of dementia	124 (5.0)	23 (8.6)	1034 (9.3)	150 (10.2)
Depression	316 (12.7)	34 (12.8)	4607 (41.3)	648 (44.0)
Severe mental illness				
Lifetime diagnosis of bipolar disorder	439 (17.7)	47 (17.7)	5170 (46.4)	755 (51.3)
Lifetime diagnosis of psychoses and schizophrenia	108 (4.3)	10 (3.8)	2318 (20.8)	317 (21.5)
Neurodevelopmental disorders				
Lifetime diagnosis of ADHD	199 (8.0)	8 (3.0)	756 (6.8)	93 (6.3)
Lifetime diagnosis of autism	445 (17.9)	55 (20.7)	2281 (20.5)	342 (23.2)

BtC, behaviours that challenge; ADHD, attention-deficit hyperactivity disorder.

Individuals who were overweight and obese also had consistently higher proportions of all three combinations of interventions than those with other physical comorbidities. High cholesterol (48.8%), constipation (49.5%) and high blood pressure (38.9%) were especially common in the group that received both interventions, as were all mental health comorbidities, notably, severe mental illnesses (i.e. bipolar disorder (51.3%) and psychosis/schizophrenia (21.5%)). The small proportion of recorded non-pharmacological interventions is likely to be attributable to underreporting.

**Fig. 2:**
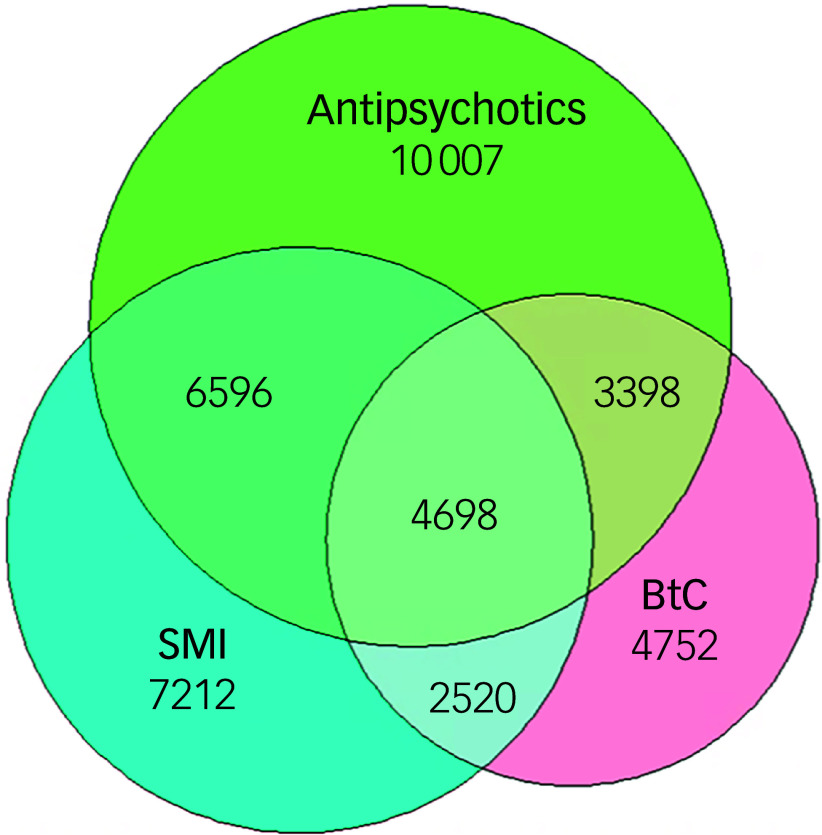
Associations between lifetime record of severe mental illness, BtC and prescription of antipsychotics. BtC, behaviours that challenge, SMI, severe mental illness.

Among the 24 699 adults with intellectual disabilities who were prescribed antipsychotics, 13 405 (54.3%) did not have a diagnosis of severe mental illness. Of the 15 368 individuals with a record of BtC, 8096 (52.7%) received antipsychotic medication, of whom 4698 (58.0%) had a diagnosis of severe mental illness. Finally, among those who were prescribed antipsychotics, 10 007 (40.5%) had no record of severe mental illness or BtC (Fig. 2).

### Outcomes

#### AHCs

**Fig. 3:**
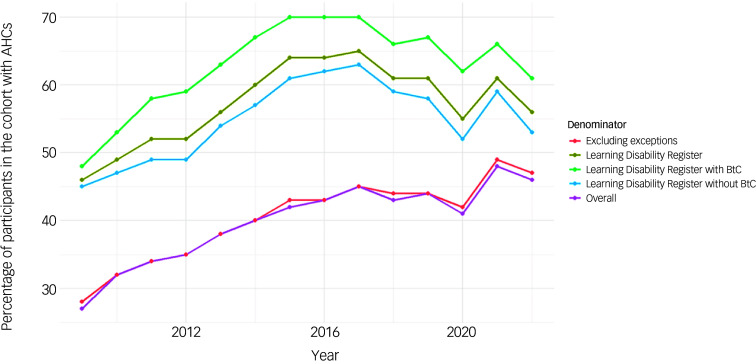
Time trends of annual health checks by BtC and Learning Disability Register status. The Register signifies the mandated recording of people with intellectual disabilities who attend primary care in England, to support better healthcare access. AHC, annual health check; BtC, behaviours that challenge.

Figure 3 shows the prevalence of AHCs in the cohort by various denominator groups: overall (i.e. everyone in the cohort), everyone except patients who had at least one exception code and no AHCs, patients on the intellectual disability register at any time and by BtC status. Exception reporting allows GP practices to exclude patients from specific indicators or clinical domains for reasons beyond their control.^18^

The percentage of individuals receiving AHCs increased from 2009 (27.4%) to 2017 (44.6%), followed by a gradual decline. A notable drop in AHCs occurred in 2020, likely attributable to the COVID-19 pandemic, but recovered after that. Trends were similar among the group with at least one exception code and no AHC.

AHC rates were consistently higher among patients who displayed BtC and were in the intellectual disabilities register. Uptake peaked at 69.8% in 2017 and despite COVID-19 disruptions, it remained above 60% through 2022. Although AHC rates for those without BtC increased between 2009 and 2017, they were lower than the BtC group.

#### GP referrals for further assessments and/or intervention

**Fig. 4:**
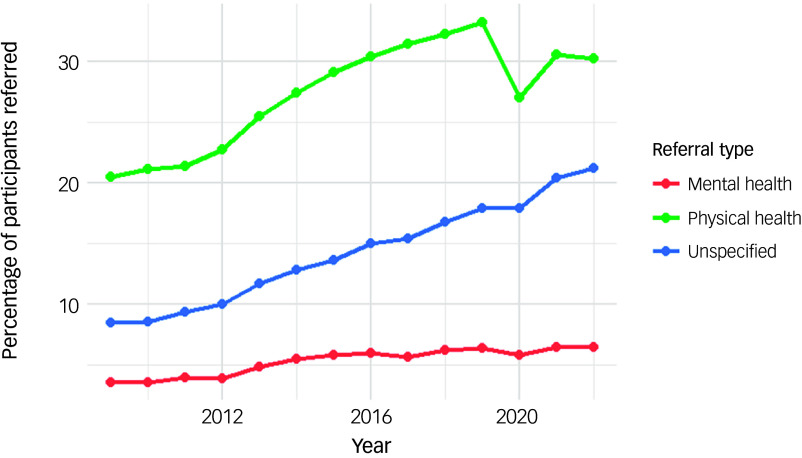
Time trends of general practitioner referrals in the cohort.

GP referrals covered data from 2009 onward. The highest proportion of GP referrals were for physical health compared with mental health and unspecified reasons. Mental health referrals were the lowest among the three types, with a range of 3.6–6.5% during the cohort period (Fig. 4).

#### All-cause accident and emergency attendance

**Fig. 5:**
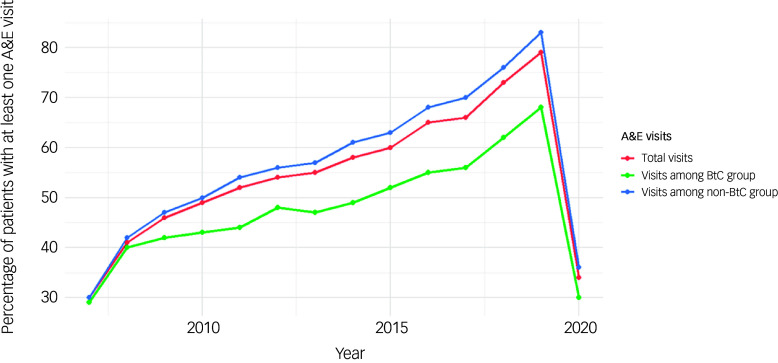
Time trends of A&E attendance by BtC status. A&E, Accident and Emergency Department; BtC, behaviours that challenge.

Figure 5 shows the percentage of patients with at least one Accident and Emergency Department (A&E) attendance per year, stratified by BtC status. Patients with BtC had consistently lower A&E usage compared with those without BtC. The highest attendance rate for patients with BtC was 68% in 2019, followed by a drop to 30% in 2020 compared with rates of attendance in patients without BtC (83% in 2019, then dropping to 36% in 2020). These rates could reflect the crossover to the new system of recording A&E attendances.

#### All-cause and cause-specific out-patient attendance and in-patient admissions

**Fig. 6:**
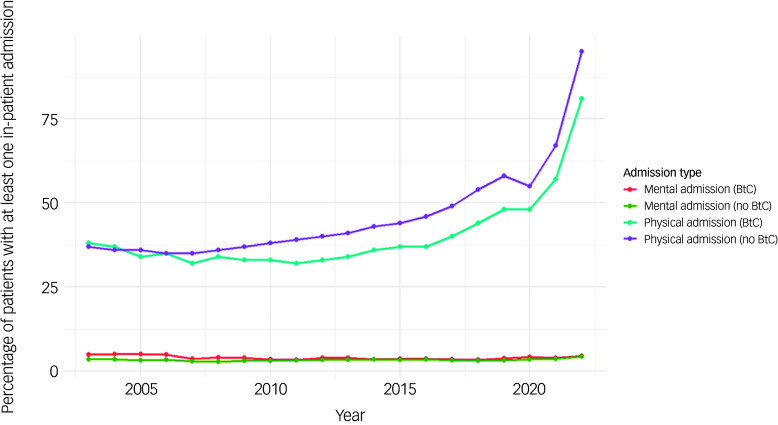
Time trends of in-patient admissions in the cohort by BtC status. BtC, behaviours that challenge.

Figure 6 displays the percentage of patients with at least one in-patient admission per year from 2003 to 2022, stratified by BtC status and reason for admission. The most notable trend is the increase in in-patient admissions for physical health reasons from 2015 onward, particularly among individuals without BtC, reaching over 80% by 2022. Those with BtC also show a rise in admissions for physical ill-health, although at a lower level.

In contrast, in-patient admissions for mental health reasons remained relatively low and stable throughout the same period. Both BtC and without BtC groups showed similar admission rates under 10% throughout the study period.

**Fig. 7:**
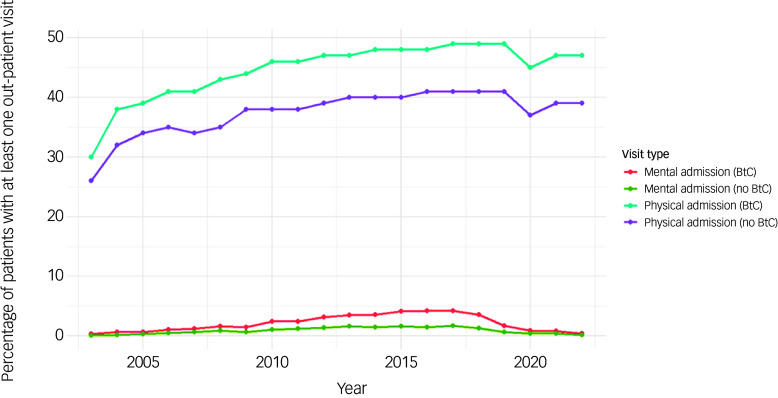
Time trends of out-patient attendance in the cohort by BtC status. BtC, behaviours that challenge.

Figure 7 displays the percentage of patients with at least one out-patient attendance per year from 2003 to 2022, stratified by BtC status and reason for visit. Physical health outpatient attendance among those with BtC rose from 30% at cohort inception to 49% by 2019. There was a slight decline after 2019, possibly reflecting the impact of the COVID-19 pandemic on in-person consultations. Physical out-patient attendance among those without BtC also increased, but the rates were consistently lower than the BtC group.

Mental health out-patient attendance rates among those with BtC gradually increased from 0.3% at cohort inception to 4.2% by 2017. Mental health out-patient attendance among those without BtC was below 2% throughout the study period.

### Associations of BtC and physical and mental health service use

Table 3 shows that adults with BtC have significantly higher odds of using mental health out-patient services (model 2: odds ratio 1.81, 95% CI 1.71–1.93). The effect is reduced after adjusting for mental and physical health comorbidities, but remains statistically significant (model 4: odds ratio 1.42, 95% CI 1.33–1.52).

**Table 3:** Models assessing the associations between BtC and health service use

	Model 1	Model 2	Model 3	Model 4
Mental health out-patient attendance, odds ratio (95% CI)				
BtC	1.82 (1.72–1.94)	1.81 (1.71–1.93)	1.47 (1.37–1.57)	1.42 (1.33–1.52)
Physical health out-patient attendance, IRR (95% CI)				
BtC	0.74 (0.71–0.77)	0.73 (0.70–0.75)	0.74 (0.71–0.76)	0.81 (0.78–0.84)
Mental health in-patient admissions, IRR (95% CI)				
BtC	1.26 (1.16–1.38)	1.22 (1.12–1.32)	1.11 (1.02–1.21)	1.19 (1.09–1.29)
Physical health in-patient admissions, IRR (95% CI)				
BtC	0.72 (0.70–0.75)	0.71 (0.68–0.73)	0.75 (0.72–0.77)	0.77 (0.74–0.79)

Notes: Model 1 is unadjusted. Model 2 is adjusted for demographic variables: gender, ethnicity, Index of Multiple Deprivation. Model 3 is adjusted for all variables in model 2 plus mental health comorbidities. Model 4 is adjusted for all variables in model 3 plus physical health comorbidities. All variables are binary (yes versus no), with the reference group set to not having the condition. Mental health out-patient models: Model 1 (*n* = 83 070, number of events: 5723); models 2 to 4 (*n* = 66 859, number of events: 5675). Physical health out-patient models: model 1 (*n* = 71 213); models 2–4 (*n* = 55 183). Mental health in-patient models: model 1 (*n* = 83 067); models 2–4 (*n* = 66 856). Physical health in-patient models: model 1 (*n* = 79 604); models 2–4 (*n* = 63 405). BtC, behaviours that challenge; IRR, incidence rate ratio.

BtC is associated with lower rates of physical health out-patient attendance (model 4: incidence rate ratio (IRR) 0.81, 95% CI 0.78–0.84), but higher rates of mental health in-patient admissions (model 4: IRR = 1.19, 95% CI 1.09–1.29), even after full adjustment.

Adults who display BtC have consistently lower rates of physical health in-patient admissions than those without BtC. The association remains statistically significant after adjusting for demographic characteristics and comorbidities (model 4: IRR = 0.77, 95% CI 0.74–0.79).

Full estimates for mental and physical health comorbidities are shown in Supplementary Table 1.

### All-cause mortality and BtC associations

**Fig. 8:**
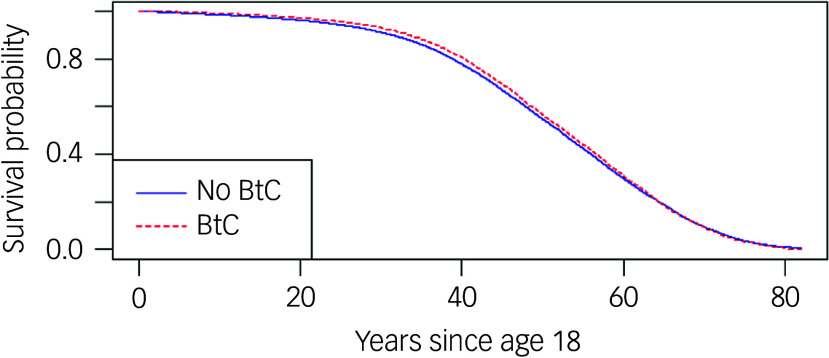
Kaplan–Meier curve by BtC status (unadjusted). BtC, behaviours that challenge.

Models 1 and 2 (Table 4) show a small but statistically significant reduction in mortality for those with BtC compared with those without BtC (hazard ratio: 0.95, 95% CI 0.92–0.98). This is supported by the Kaplan–Meier curve (Fig. 8). Models 3 and 4, after adjusting for mental and physical health comorbidities, show a weaker and non-significant association, suggesting that health comorbidities explain much of the difference. Despite the high proportions of bipolar disorder, psychoses and schizophrenia (Table 1), severe mental illness is less likely to influence mortality (model 4: hazard ratio: 0.93, 95% CI 0.90–0.96). In contrast, neurodevelopmental conditions, anxiety, depression and epilepsy are more likely to influence mortality (Table 4).

**Table 4:** Models assessing the association between BtC and mortality

	Model 1	Model 2	Model 3	Model 4
Mortality, hazard ratio (95% CI)				
BtC	0.95 (0.92–0.98)	0.95 (0.92–0.98)	0.99 (0.95–1.02)	0.97 (0.93–1.00)
Autism	–	–	1.08 (1.02–1.15)	1.08 (1.01–1.15)
ADHD	–	–	1.39 (1.21–1.59)	1.38 (1.21–1.57)
Severe mental illness	–	–	0.89 (0.86–0.92)	0.93 (0.90–0.96)
Anxiety	–	–	0.96 (0.92–1.01)	1.04 (0.99–1.09)
Depression	–	–	0.99 (0.95–1.03)	1.02 (0.98–1.07)
Dementia	–	–	0.92 (0.88–0.96)	0.87 (0.84–0.90)
Overweight/obese	–	–	–	0.82 (0.79–0.84)
Type 2 diabetes	–	–	–	0.93 (0.89–0.96)
High blood pressure	–	–	–	0.79 (0.77–0.81)
High cholesterol	–	–	–	0.87 (0.85–0.90)
Severe mobility difficulties	–	–	–	0.90 (0.88–0.93)
Epilepsy	–	–	–	1.75 (1.70–1.80)
Constipation	–	–	–	0.95 (0.93–0.98)

Model 1 is unadjusted. Model 2 is adjusted for demographic variables: gender, ethnicity, Index of Multiple Deprivation. Model 3 is adjusted for all of the variables in model 2 plus mental health comorbidities. Model 4 is adjusted for all of the variables in model 3 plus physical health comorbidities. All variables are binary (yes versus no), with the reference group set to not having the condition. Model 1 (*n* = 83 166, number of events: 22 933); Models 2–4 (*n* = 66 954, number of events: 22 208). BtC, behaviours that challenge; ADHD, attention-deficit hyperactivity disorder.

## Discussion

### Main findings

Adults with intellectual disabilities who display BtC had markedly higher rates of both physical and mental health comorbidities and autism. They also had more recorded psychotropic medications and non-pharmacological interventions compared with those without BtC. A total of 40.5% of those who were prescribed antipsychotics had no recorded severe mental illness or BtC. BtC was not independently associated with increased mortality, but comorbid neurodevelopmental disorders, common mental disorders and epilepsy raised mortality risk. As expected, BtC predicted increased use of both in-patient and out-patient mental health services, but, counterintuitively, not physical health services. The study period encompasses the COVID-19 pandemic, during which adults with intellectual disabilities and neurodevelopmental conditions experienced disproportionate disruption to health and social care, increased social isolation and reduced access to routine services. As cohort eligibility ended on 31 December 2022 to allow for at least a year of follow-up, the data includes the pandemic and immediate post-pandemic period rather than full recovery. Pandemic-related changes in service access and recording practices may therefore have influenced observed patterns of service use, morbidity and prescribing. These effects could have contributed to unmet physical health needs and increased reliance on psychotropic medication during this period.

### Strengths and limitations of the study

This is the first study, to our knowledge, to explore trajectories of health outcomes in a high-need population, using a representative, population-based sample with linked secondary care data spanning two decades – allowing for robust longitudinal analysis. Use of clinician-verified code lists for BtC and comorbidities, alongside a wide range of covariates, strengthened the study’s validity. BtC have major implications for individuals with intellectual disabilities and their families and therefore, investigating long-term prognosis can support personalised care, improve symptom recording and management, and inform service planning.

There are some limitations to the study that should be noted. Clinical coding in electronic health records may not fully capture BtC or mental illness diagnoses, introducing potential misclassification. The high rates of severe mental illness in our sample could have been due to this or the presence of false-positives.

The higher prevalence of physical comorbidities among individuals receiving psychotropic medication, particularly high cholesterol, overweight or obesity, and high blood pressure, should be interpreted with caution, as these may partly reflect iatrogenic effects rather than baseline physical health differences alone. As this was an observational study using routinely collected data, we were unable to determine the temporal relationship between psychotropic medication use and onset of these conditions. Higher rates of metabolic conditions were also observed among individuals receiving non-pharmacological interventions only, which could reflect residual confounding, such as pre-existing physical health needs that prompted intervention, rather than an effect of the intervention itself.

The significant finding of a reduction in mortality among those with BtC in the unadjusted models may be influenced by immortal time bias,^19^ as individuals could only receive a BtC code if they survived long enough to obtain it, thus gaining additional survival time. Additionally, non-pharmacological interventions were likely underrecorded, as these are often not shared with GPs and research indicates that psychoeducation is the most frequently offered support in mental health services among those with intellectual disabilities, with 36% offering no named intervention in the past year.^20^ As not all practices had HES linkage, it limited the secondary care analysis sample. Also, we could not stratify by level of intellectual disabilities because of inconsistent recording. Lastly, the observational design limits causal inference, with residual confounding possible.

### Findings in context

The lower contact of physical health service use among individuals with BtC is concerning, especially given the substantial rates of psychotropics prescribing and the relatively higher offer of AHCs.^21^ Persistent barriers such as difficulty accommodating individuals with BtC in physical health settings, diagnostic overshadowing^22^ or the episodic nature of BtC may contribute to this disconnect between need and service access.

Our results reflect a high burden of mental ill-health and autism among adults with intellectual disabilities – particularly those with BtC – consistent with prior research.^23^ International studies.^24,25^ similarly show sustained psychiatric morbidity in this group. Although antipsychotic prescribing without a recorded severe mental illness is still prevalent, it has declined compared with earlier reports,^7,26^ possibly reflecting improved severe mental illness recognition or changing prescribing thresholds.

Contrary to earlier CPRD studies showing elevated mortality linked to intellectual disabilities,^27^ we did not find BtC to be independently associated with higher mortality. The earlier cohort (2010–2014) predated key care improvement initiatives such as the STOMP campaign (Stopping the Overmedication of People with Intellectual Disabilities),^28^ which may have contributed to more recent health gains.

The long study period (2003–2023) spans substantial changes in clinical practice and policy relating to the care of adults with intellectual disabilities. There has been increasing awareness of physical and mental health inequalities, greater recognition of neurodevelopmental disorders, evolving thresholds for diagnosing severe mental illness and heightened scrutiny of psychotropic prescribing. Changes in recording practices and service awareness may therefore partly explain the high prevalence of psychiatric and neurodevelopmental diagnoses observed. Our findings should thus be interpreted as reflecting both true clinical burden and advancing recognition and documentation of health needs in this population.

### Possible explanations and implications for clinicians and policy makers

The growing prevalence of clinical comorbidities in the cohort over time is creating a chronic disease burden for people with intellectual disabilities. Importantly, BtC drive mental health out-patient attendance and hospital admissions, but not physical health input, despite national standards recommending physical comorbidity investigation as an early step in the diagnostic pathway.^21^ This discrepancy suggests a misalignment between policy and practice, and calls for further tailoring of approaches for those with complex behavioural and health needs to improve personalised care for a costly and common mental health condition.

Applying the International Classification of Functioning, Disability and Health (ICF) framework^29^ helps conceptualise BtC not merely as a symptom of mental illness, but as the outcome of interactions between impairments, environmental barriers and personal factors. Adults with BtC often face chronic illness and multimorbidity, which heightens vulnerability and complicates care. The high rate of mental health service use may reflect a focus on impairment-based care, whereas the lack of physical health engagement suggests that other domains of functioning (e.g. participation and access) are not being consistently addressed. The ICF model supports a more holistic and context-sensitive approach, promoting integrated care that combines physical health monitoring, mental health support and non-pharmacological strategies. Such approaches are crucial to achieving equitable health outcomes and enhancing quality of life for people with BtC.

### Unanswered questions and future research

Recent policy changes such as the removal of AHC targets from the Quality and Outcomes Framework^30^ risk de-prioritising these checks, especially in overstretched practices. Additionally, the absence of references to hard-to-reach populations in the latest National Health Service 10-Year Plan^31^ raises concerns that hard-won progress in the care of people with intellectual disabilities, including those who display BtC, may be reversed. Enhanced data on BtC severity, frequency, context and interventions received promoted by the specialist community services would aid in stratifying risk and guiding preventative strategies. Future research should monitor how policy shifts affect access, treatment delivery and health equity. This is essential to determine whether reforms are translating into meaningful improvements in outcomes for this high-risk and underserved population.

## Supporting information

10.1192/bjo.2026.11043.sm001 Jagtiani et al. supplementary material Jagtiani et al. supplementary material

## Data Availability

CPRD data are not publicly available. However, any other data related to the study (e.g. code lists) can be made available from the corresponding author, A.H., upon reasonable request.

## Supplementary material

The supplementary material is available online at https://doi.org/10.1192/bjo.2026.11043
